# P-81. A Decade of Culture-Negative Native Vertebral Osteomyelitis: Insights from the Mayo Clinic

**DOI:** 10.1093/ofid/ofae631.288

**Published:** 2025-01-29

**Authors:** Rita Igwilo-Alaneme, Said El Zein, Ryan B Khodadadi, Francesco Petri, Omar Mahmoud, Elie Berbari

**Affiliations:** Mayo Clinic, Rochester, Minnesota, Rochester, Minnesota; Mayo Clinic, Rochester, Minnesota; Mayo Clinic, Rochester, Minnesota; Mayo Clinic, Rochester, Minnesota, Rochester, Minnesota; Mayo Clinic, Rochester, Minnesota, Rochester, Minnesota; Mayo Clinic, Rochester, Minnesota

## Abstract

**Background:**

Culture-negative native vertebral osteomyelitis (CN-NVO) is increasingly common, rising from 0.3 to 1.8 cases per 100,000 between 1995 and 2008. The lack of microbial identification complicates optimal treatment and creates prognostic uncertainties. We describe our decade-long clinical experience with CN-NVO, focusing on patient characteristics, treatment, and outcomes.

Patient Characteristics

**Figure:**
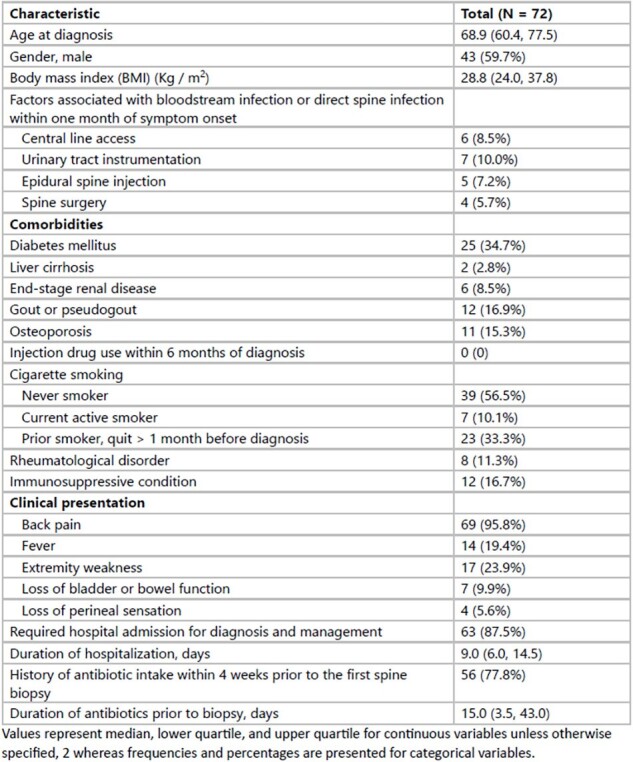

**Methods:**

We retrospectively analyzed adult patients with radiographic evidence of NVO between January 1, 2011, and July 31, 2021. CN-NVO required clinical indicators such as back pain and fever, elevated inflammatory markers, and negative cultures from at least one spine biopsy. Patients with infected instrumentation were excluded.

Diagnostic Evaluation

**Figure:**
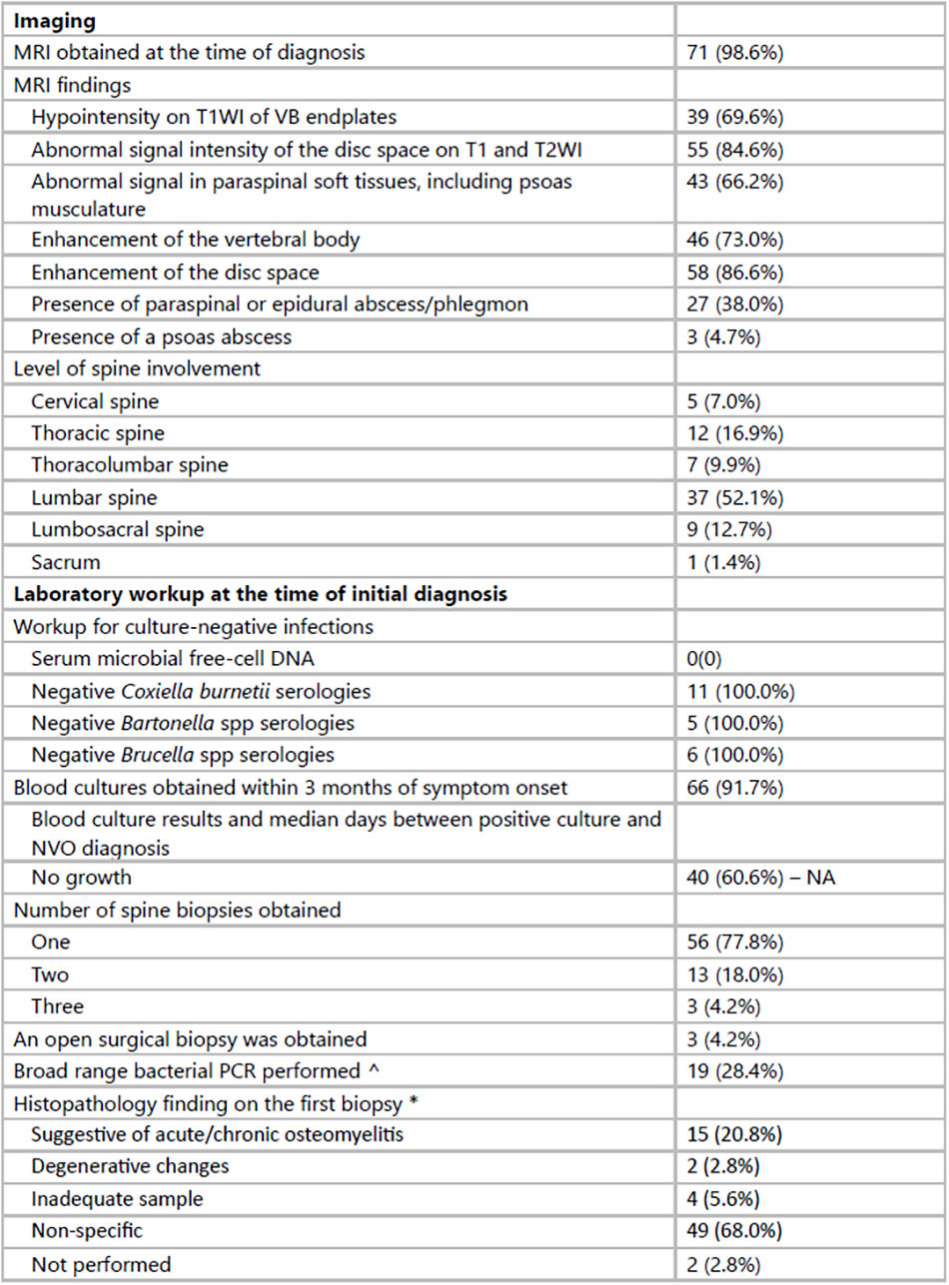

Values represent median, lower quartile, and upper quartile for continuous variables unless otherwise specified, 2 whereas frequencies and percentages are presented for categorical variables.

^ All results returned negative

*Biopsies number two and three did not result in additional findings or change clinical management.

**Results:**

Seventy-two patients were included. Their characteristics are presented in Table 1. Most (77.8%) had received antibiotics within the 4 weeks preceding the biopsy. Disc space enhancement on MRI was the primary finding, with the lumbar spine being the most frequently involved (Table 2). Blood cultures were positive in 39.4% of the patients within 3 months of symptom onset. Streptococcus spp. (n=9, median time between positive culture and diagnosis: 6.5 days [IQR 2.25, 26.75]) and Staphylococcus aureus (n=7, 40 days [IQR 37.5, 51.5]) were most common. Definitive antibiotics were administered in 98.6% of the cases, primarily IV β-lactam + vancomycin. Oral antibiotics (PO) were used in 46.5% of the patients, primarily as step-down therapy (91%) (Table 3). Surgery was required in 8.5% of the patients for abscess drainage or relief of spinal cord compression. Treatment failure occurred in 9.4% of patients, and 37.5% died during the median follow-up of 568.5 days, with two deaths related to the infection.

Treatment and Outcomes

**Figure:**
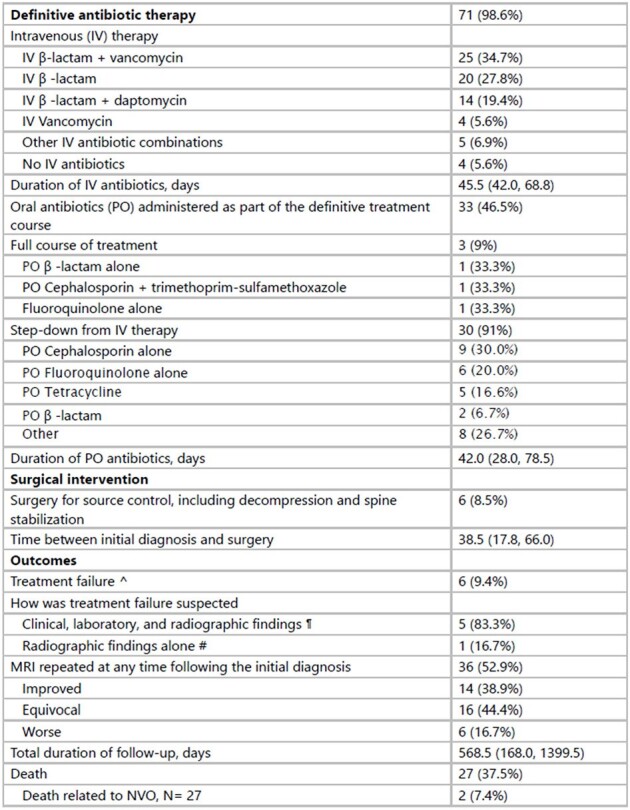

^ defined as requiring another course of antibiotics and/or surgery for suspected ongoing infection

¶ Clinical findings include back pain or fevers; laboratory findings indicate abnormal or uptrend in inflammatory marker values

# Defined as MRI findings concerning ongoing or worsening infection as per the interpretation of the Radiologist, Infectious Disease physician, and/or Orthopedic Surgeon

**Conclusion:**

Patients with CN-NVO often require prolonged antibiotic therapy, typically combination regimens, with favorable treatment outcomes. Our treatment failure rate is similar to other studies, although comparability may be limited by heterogeneity in definitions. Mortality rates in the range of 6 and 36% have been reported, with the higher end driven mostly by comorbidities. Enhanced diagnostics and standardized treatment protocols are needed to further improve patient outcomes.

**Disclosures:**

**All Authors**: No reported disclosures

